# Burnout, stress and Type D personality amongst hospital/emergency physicians

**DOI:** 10.1007/s00420-021-01766-z

**Published:** 2021-10-15

**Authors:** Francis Somville, Gerry Van der Mieren, Harald De Cauwer, Peter Van Bogaert, Erik Franck

**Affiliations:** 1 Department of Emergency Medicine, University of Antwerp, UZA, Wilrijkstraat 10, 2650 Edegem, Belgium; 2 Department of Emergency and Traumatology, AZ St Dimpna, J.B Stessensstraat 2, 2440 Geel, Belgium; 3 Department of Neurology, AZ St Dimpna, J.B Stessensstraat 2, 2440 Geel, Belgium; 4 Department of Nursing and Midwifery Sciences, Centre for Research and Innovation inCare (CRIC), Campus Drie Eiken, Universiteitsplein 1, 2610 Wilrijk, Belgium

**Keywords:** Burnout, Occupational stress, Type D personality, Hospital physicians, Emergency physicians

## Abstract

**Introduction:**

In previous studies, physicians have been identified as a high-risk group for burnout.

Although the work environment has received more attention than specific determinants of personality traits, the latter might contribute to burnout.

Study objective.

We aimed to investigate the association of Type D personality, job and organizational determinants with burnout, stress and work engagement as outcome factors among emergency physicians and hospital physicians working in intensive care and surgery departments.

We specifically focused on our group of emergency physicians.

**Methods:**

In this cross-sectional study, self-report questionnaires were distributed via social media using a specific survey link to 531 Belgian hospital physicians working at the Emergency Department, Intensive Care, and Surgery Department between October 21, 2018, and April 11, 2019. The survey instrument included questions about sociodemographic characteristics, job characteristics, organizational factors, job satisfaction, social support by supervisors and colleagues (Leiden Quality of Work Questionnaire for Medical Doctors) and Type D personality (Distress Scale-14) and as outcomes burnout (Oldenburg Burnout Inventory) and work engagement (Utrecht Work Engagement Scale). A multiple regression analysis was used to examine the associations between the determinants and each of the outcomes with emergency physicians as the study population.

**Results:**

Eligible data were available for 436 questionnaires and involved 212 emergency physicians, 162 other hospital physicians (Intensive Care and Surgery Department) and 62 residents concerning both groups of physicians. Type D personality ranged from 28.5 to 29.1% in emergency physicians and other hospital physicians. Additionally, even after correcting for job-related and organizational factors, emergency physicians with Type D personality were seven times more likely to have a high risk for burnout.

**Conclusion:**

As a result, this study offers a new perspective on the associations between burnout, stress and Type D personality. Type D personality might be a personality-related risk factor for burnout among emergency physicians. Therefore, we recommend enhanced prevention measures that take into account this individual factor in the further development of coaching programs. Improving the professional well-being of emergency physicians is necessary, especially in the scope of the recent COVID-19 pandemic, which has put a high demand on acute and emergency care departments.

## Introduction

In the last decade, awareness of occupational stress and burnout among physicians has increased. Physician burnout has reached epidemic levels, with studies demonstrating prevalence ranging from 43.9% to near 54%. (Dyrbye et al. 2008; Shanafelt et al. 2009, 2015; West et al. 2011) In particular, emergency physicians are at risk because of emotional, physical and intellectual challenges. (Arora et al. 2013) Burnout was defined by Maslach et al. as a psychological syndrome that has three dimensions: emotional exhaustion, depersonalization (disengagement) and reduced personal accomplishment. (Maslach et al. 2008) In some studies, a clear significant relationship between observed patient outcomes and physician burnout was found. (Mangory et al. 2021) Several pathogenic work-related factors have been identified in the course of burnout (Schonfeld et al. 2018), yet personality traits were found to moderate the relationship between work-related factors and burnout (Geuens et al. 2017). Work stress factors in physicians are often multifactorial. Physicians are at high risk for burnout development. (Wal et al. 2018).

Emergency physicians are especially prone to work-related traumatic events, hectic stressful working conditions, occupational risks, lack of social support, psychological problems, subjective fatigue, somatic complaints, and conflicts with other physicians (Somville et al. 2016). A wide range of studies have been conducted on work-related and organizational factors and highlighted the working relationship between physicians and nursing staff, ED supervisors, and hospital management (Doef 1999; Doef and Maes 1999). Although all physicians in the ED are exposed to the same job-related and organizational factors, individual characteristics such as personality traits of the physicians may also play a crucial role in the development of burnout (Wal et al. 2018).

This study focused on the impact of individual determinants contributing to the development of burnout. In the current study, these individual determinants were studied using the limited body of evidence of Type D personality (D stands for Distressed) research method. It is a relatively stable personality trait and consists of a combination of negative affectivity and social inhibition (Denollet 2005; Borkoles et al. 2018; Polman et al. 2010). Those who experience a high grade of negative affectivity have a tense feeling, loss of personal contact and an uneasy feeling when interacting with other people (Williams and Wingate 2012; Demerouti et al. 2001). This social inhibition contributes to the concept of negative affectivity (Muraven and Baumeister 2000). Mols and coworkers indicate that individuals with a Type D personality are more likely to experience their environment as stressful but are less likely to ask for help (Mols and Denollet 2010). Type D personality has been identified as a determining factor for mental health problems, long-lasting stress periods, and burnout (Denollet 2005). The correlation between Type D personality and burnout has been validated in European and Canadian general populations, as well as in Dutch anesthesiologists (Wal et al. 2018; Polman et al. 2010; Mols and Denollet 2010; Wal et al. 2016). However, these studies only investigated personality factors and did not include job-related and organizational factors. Personality traits account for nearly 60% of the variance in burnout (Denollet 2005; Bianchi 2018; Scanlan and Still 2019). In the current COVID-19 pandemic, the impact of various determinants on mental health is increasing even more among health care workers. Multiple determinants should be considered when approaching and making considerations to offer support to colleagues in these COVID-19 times (Chirico et al. 2021; Chirico and Nucera 2020; Chirico et al. 2020; Chirico and Magnavita 2020).

We aimed to investigate the association between Type D personality and burnout in emergency physicians and compare this personality trait with hospital physicians working in intensive care and surgery departments. The unique approach of our study is to investigate the influence of type D personality on the job content of ED physicians.

## Methods

### Study design and sample collection

#### Procedures and ethical aspects

**Fig. 1:**
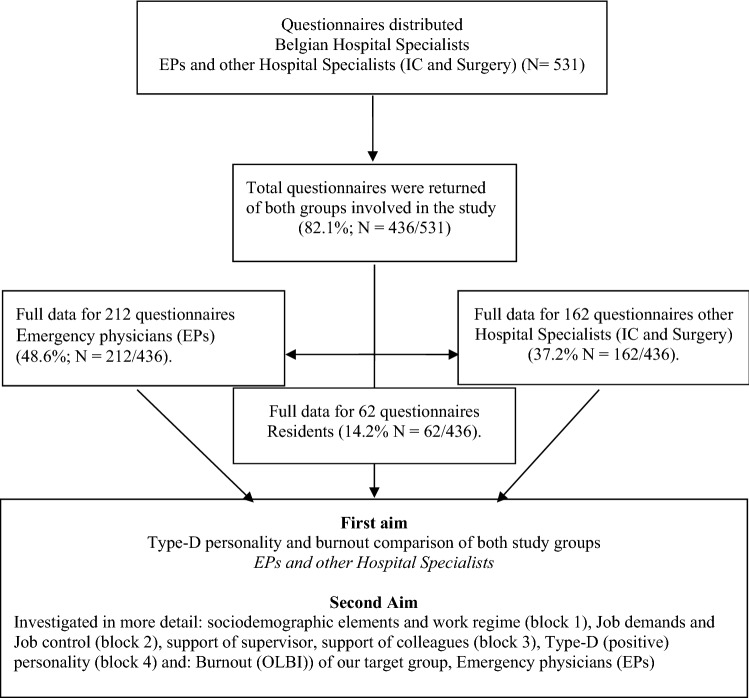
Flow diagram of the strategy used to collect survey data and develop the strategy for the study

Study approval from the Ethical Committee of St. Dimpna Hospital, Geel was obtained (EC OG099 nr:709). Confidentiality was guaranteed to all participants. Informed consent was signed by each respondent before data collection. As clarified in the flow diagram (Fig. 1).

#### Data collection

During this cross-sectional study, data were collected using self-report questionnaires distributed to Belgian hospital physicians (ED, IC, surgery). A reminder was sent 1 month after the initial invitation. To guarantee confidentiality, questionnaires could be returned in a closed envelope or were protected online by a personal code. Of the 531 questionnaires that were sent, eligible data were available for 436 questionnaires (response rate 82.1%; *N* = 436/531). The survey instrument included questions such as sociodemographic characteristics, job characteristics, organizational factors, job satisfaction, social support by supervisors and colleagues (LQWQ-MD) and Type D personality (DS-14) and outcomes burnout (OLBI) and work engagement (UWES). The departments and physicians surveyed between October 21, 2018, and April 11, 2019, and the respondents were working in fully specialized ER. Hierarchical multiple regression analyses were used to examine the association between the determinants and each of the outcomes.

#### Measuring instruments

Several instruments that measure feelings of burnout have been developed, but only a few have been validated in physicians. Demerouti introduced the Oldenburg Burnout Inventory (OLBI), which is the modified and validated version of the Maslach Burnout Inventory Human Service Survey (MBI-HSS), which can be applied specifically to physicians. In addition, we used the Utrecht Work Engagement Scale (UWES) to measure engagement, which is the opposite of burnout (Maslach and Leiter 1997, 2008). Seppälä and Schaufeli described the validity of the work engagement measure (Seppälä et al. 2008; Schaufeli and Bakker 2004).

The questionnaire consisted of validated instruments concerning Type D personality (DS-14), burnout (OLBI) and work engagement (UWES). A fourth validated instrument, the Leiden Quality of Work Questionnaire for physicians (LQWQ-MD), assessed job-related and organizational factors and was added to correct for the influence of these factors. These validated instruments were supplemented with demographical questions and job characteristics.

Type D personality was measured using the DS-14 questionnaire, which assesses negative affectivity and social inhibition. Example items for measuring negative affectivity included statements such as "I often feel unhappy" and "I am often in a bad mood". The score for social inhibition was determined through statements such as "I find it hard to start a conversation" and "I often feel inhibited in social interactions". For both subscales, participants were asked to rate to what degree the statements were true for them on a scale from 0 to 4. Type D personality was diagnosed when scores reached 10 or more on both the negative affectivity scale and the social inhibition scale. In the present study, Cronbach's alpha coefficient was 0.87, indicating good reliability (Denollet 2005).

Burnout was evaluated using the OLBI (the Oldenburg Burnout Inventory) (Demerouti et al. 2001). The instrument consists of 16 items that measure the frequency of the main burnout symptoms on a 4-point scale across two dimensions: emotional exhaustion and disengagement. For the emotional exhaustion dimension, example statements included "During my work, I increasingly feel emotionally exhausted". For the disengagement dimension, example statements included “lately I tend to think less at work and do my job almost mechanically”. Dutch cutoff values specified for physicians were used because Belgian cutoff values were not available. Using these cutoff scores, burnout was indicated by a score ≥ 2.25 for exhaustion and a score ≥ 2.10 for disengagement (Westwood et al. 2017). Participants responded by choosing one of four responses from “strongly agree” to “strongly disagree”. For calculating mean scores for each of the two components, items were reversed when necessary so that a higher score indicated more exhaustion or disengagement. In the present study, the Cronbach’s alpha coefficient (α) was 0.83 for exhaustion and (α) 0.87 for disengagement. The binary variable indicating whether the participant was suffering from problematic burnout was created using the cutoff scores on the OLBI, which correspond to those on the MBI-HSS found to predict physician-diagnosed burnout.

Work engagement was assessed using the Utrecht Work Engagement Scale (Seppälä et al. 2008). The UWES-9 work engagement score is the translated and validated version of the UWES (Schaufeli and Bakker 2004), which can be applied specifically to physicians. The instrument consists of 9 items that measure the frequency of engagement. Cronbach’s alpha coefficient for this scale was α = 0.92. The items of the UWES are grouped into three subscales: vigor (α = 0.86; 3 items) (e.g., ‘At my work, I feel that I am bursting with energy’); dedication (α = 0.86; 3 items) (e.g., ‘I am enthusiastic about my job’); and absorption (α = 0.75; 3 items) (e.g., ‘I am immersed in my work’). All items were scored on a 7-point rating scale, ranging from 0 (never) to 6 (daily). As a result of the moderate intercorrelations of the subscales, only the total score was used in the present study. High scores are indicative of work engagement.

The Leiden Quality of Work Questionnaire for physicians (LQWQ-MD) instrument was used to assess job-related and organizational factors (Doef and Maes 1999). For the purpose of this study and in accordance with the guidelines of the LQWQ-MD (Doef 1999), the total score for the subscales Work and Time Demands and Physical Demands was used to measure job demands (α = 0.79; 9 items); the total score for the subscales Skill Discretion and Decision Authority was used as a measure of job control (α = 0.78; 6 items). Social support by the supervisor and colleagues was measured using two subscales of the validated LQWQ-MD (Doef 1999). Social support supervisor (α = 0.93; 4 items) measures perceived social support by the supervisor. Social support colleagues (α = 0.89; 4 items) measured perceived instrumental and emotional support by colleagues. Job satisfaction was measured using the job satisfaction subscale (α = 0.87; 3 items) of the validated LQWQ-MD (Doef 1999). Turnover intention was measured by the turnover subscale (α = 0.88; 3 items) of the validated LQWQ-MD (Doef and Maes 1999). The outcome work-home interference (α = 0.77; 4 items) was measured by a subscale of the LQWQ-MD (Doef 1999).

### Data analysis

SPSS version 27.0 was used to analyze the data. Only parametric statistics were applied. One-way ANOVA and chi-square tests were selected for between-group comparisons of Type D personality, stress and burnout within the hospital physicians. In addition, Pearson's correlation was selected to calculate the relation between the total Type D score and the burnout dimensions. Furthermore, we conducted a multiple logistic regression analysis with age(per 1 year), gender(male 1 versus female 0), work regime(full time 1 versus part time 0), job demands(1 point per increase), job control(1 point per increase), support supervisor(1 point per increase), support colleagues(1 point per increase), Type D personality(positive 1 versus negative 0) as explanatory variables and risk of burnout among emergency physicians (yes 1 versus no 0) as dependent variables. A statistical significance level of P < 0.05 and 95% confidence interval was set.

## Results

Full data were available for 436 participants (response rate 82.1%; *N* = 436/531). Women represented 56.2% of the total study sample, and the mean age of respondents was 37 years. The majority were emergency physicians (48.8%), with a mean seniority years working in the emergency department of 9.9 years and 90.9% working alternating shifts of 12 h (full time). (Table 1).

**Table 1:** Personal and job-related characteristics

Characteristics		All *N* = 436%/*Years*	Other hospital specialists *N* = 162%/Years	EPs *N* = 212%/Years
Women		56.2	56.0	54.2
Age		*37.4*	40.5	*38.9*
Marital status				
	Married/cohabitating	65.0	73.5	72.6
	Single/living alone	35.0	26.5	27.4
Seniority		*8.0*	*8.1*	*9.9*
Specialty area				
	Hospital specialist (IC/surgery)	37.2		
	Emergency physicians (Eps)	48.8		
	Master medicine	14.0		
Function				
	Emergency fellow			63.2
	Emergency resident			36.8
Work-regime 12 h				
	Alternating shifts(full time)	72.3	80.1	90.9
	Day or night shifts only(part time)	27.2	19.9	9.1

Within the total study sample, the average prevalence of burnout was 58.0% based on the cutoff values (Demerouti et al. 2001), and the average UWES score was 3.87 (SD 1,13). The exhaustion, disengagement and burnout rates of the emergency physicians (*N* = 212) were 75.7% (mean score 2.60 SD 0.43), 67.4% (mean score 2.29 SD 0.47) and 61.6% (mean score 2.45 SD 0.49), respectively, as well as a mean UWES score of 3.91 (SD 1.06) (Table 2).

**Table 2:** Prevalence of burnout/UWES (work engagement) hospital specialists and emergency physicians

Burnout/Work engagement OLBI	Total sample *N* = 436 % (SD)	Other Hospital specialists *N* = 162 % (SD)	Emergency physicians *N* = 212 % (SD)
Exhaustion	76.0 (0.45)	67.2 (0.45)	75.7 (0.43)
Disengagement	66.0 (0.47)	72.5 (0.47)	67.4 (0.47)
Burnout score	58.0 (0.49)	58.0 (0.50)	61.6 (0.49)
UWES	mean (SD)	mean (SD)	mean (SD)
Work Engagement score	3.90 (1.06)	3.87 (1.13)	3.91 (1.06)

In this study sample, the average prevalence of Type D personality among emergency physicians was 28.5% (Table 3).

**Table 3:** Prevalence of Type D and burnout/UWES (Work engagement) among other hospital physicians and emergency physicians

Type D personality	Burnout/UWES	Other hospital specialists *N* = 162%	Emergency physicians *N* = 212%
Type D positive	Type D (SD)	**29.1 (0.47)**	**28.5 (0.45)**
Type D negative		70.9	71.5
Type D positive	with Burnout (SD)	**75.9 (0.49)**	**86.7 (0.48)**
Type D negative	with Burnout	27.0	31.8
Type D positive	with UWES (SD)	**3.40 (1.06)**	**3.29 (1.11)**
Type D negative	with UWES	4.46	4.57

Regarding the relationship between Type D personality and burnout in emergency physicians, a strong positive correlation was observed (r = 0.41, P < 0.001). Job demands and burnout also showed a positive correlation (r = 0.31, P < 0.001), job control (r = -0.32, P < 0.001), and social support of colleagues (r =—0.20, P < 0.001). The UWES score had a strong negative correlation with Type D personality.

**Fig. 2:**
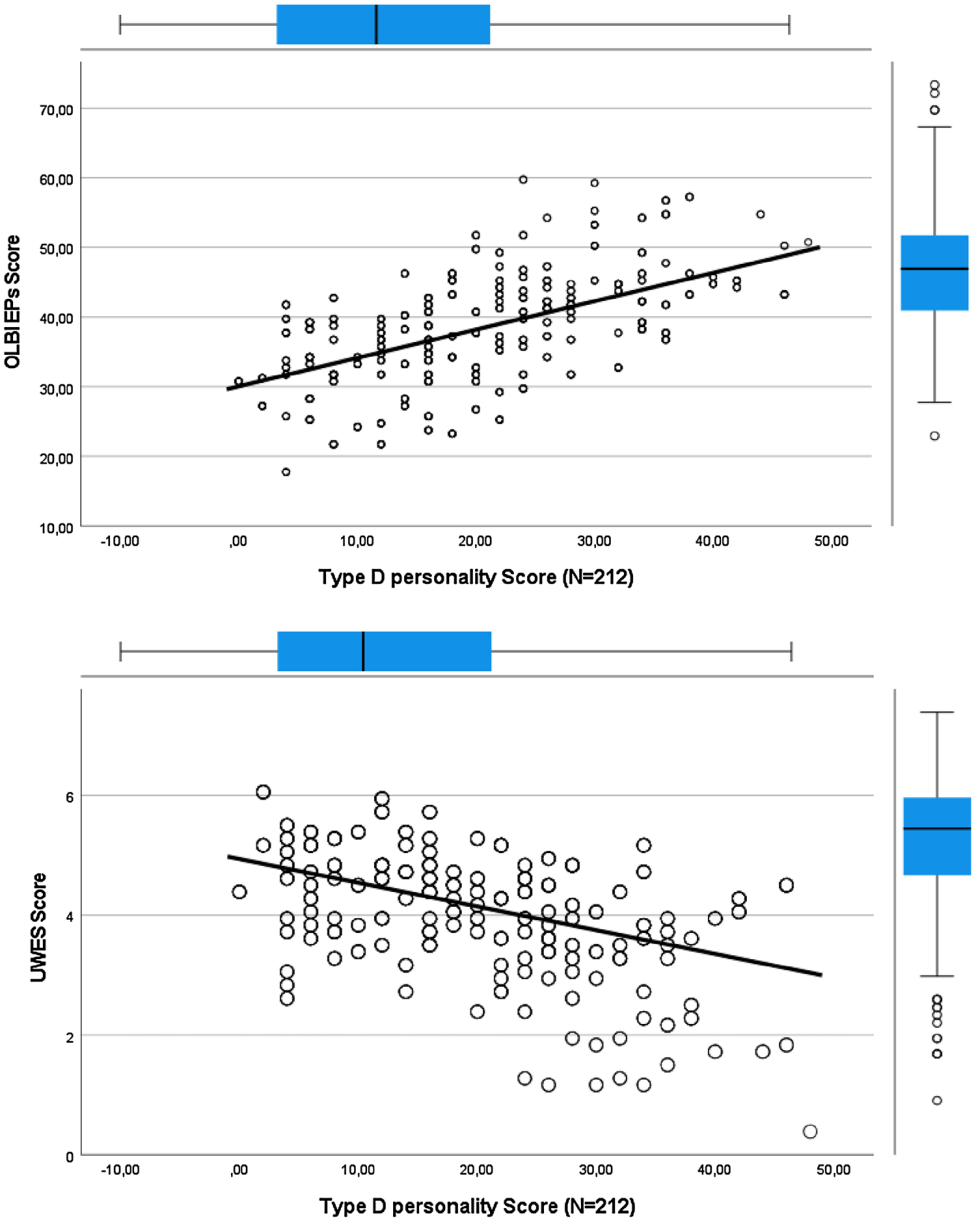
Correlation burnout (OLBI) and Type D personality, UWES and Type D personality

In addition to the vulnerability of type D personality, occupational factors such as job-related and organizational factors influenced the risk of burnout. As shown in Table 4, we found a strong positive correlation between Type D personality and job demands and work home interference. Job control, support of colleagues and job satisfaction had a strong negative correlation with Type D personality (Table 4). The correlations between Burnout and Type D, UWES and Type D are more clarified in the diagrams below Table 4 (Fig. 2).

**Table 4:** Correlations measured in emergency physicians

Measure α		1	2	3	4	5	6	7	8	9	10	11	12
Age	–	–											
Seniority	–	0.876^**^	–										
Job demands	0.79	0.160^*^	0.164^*^	–									
Job control	0.78	− 0.141	− 0.139	− 0.394^**^	–								
Support supervisor	0.93	− 0.202^**^	− 0.252^**^	− 0.129	0.389^**^	–							
Support colleagues	0.89	− 0.126	− 0.117	− 0.089	0.200^**^	0.463^**^	–						
Job satisfaction	0.87	− 0.098	− 0.123	− 0.441^**^	0.585^**^	0.423^**^	0.386^**^	–					
Turnover	0.88	− 0.116	− 0.058	0.315^**^	− 0.450^**^	− 0.398^**^	− 0.259^**^	− 0.618^**^	–				
Work-home interference	0.77	− 0.025	0.032	0.541^**^	− 0.312^**^	− 0.112	− 0.105	− 0.397^**^	0.349^**^	–			
UWES	0.91	− 0.177^*^	− 0.155^*^	− 0.231^**^	− 0.443^**^	0.400^**^	0.330^**^	0.587^**^	− 0.274^**^	0.216^**^	–		
OLBI Burnout	0.91	0.068	0.059	0.314^**^	− 0.320^**^	− 0.115	− 0.201^**^	− 0.333^**^	0.228^**^	0.264^**^	− 0.434^**^	–	
Type D personality	0.84	0.008	0.035	0.216^**^	− 0.176^*^	− 0.114	− 0.178^*^	− 0.264^**^	0.107	0.259^**^	− 0.440^**^	0.413^**^	–

**Correlation is significant at the 0.01 level (2-tailed)

*Correlation is significant at the 0.05 level (2-tailed)

α = Cronbach’s alpha coefficient

**Fig. 3:**
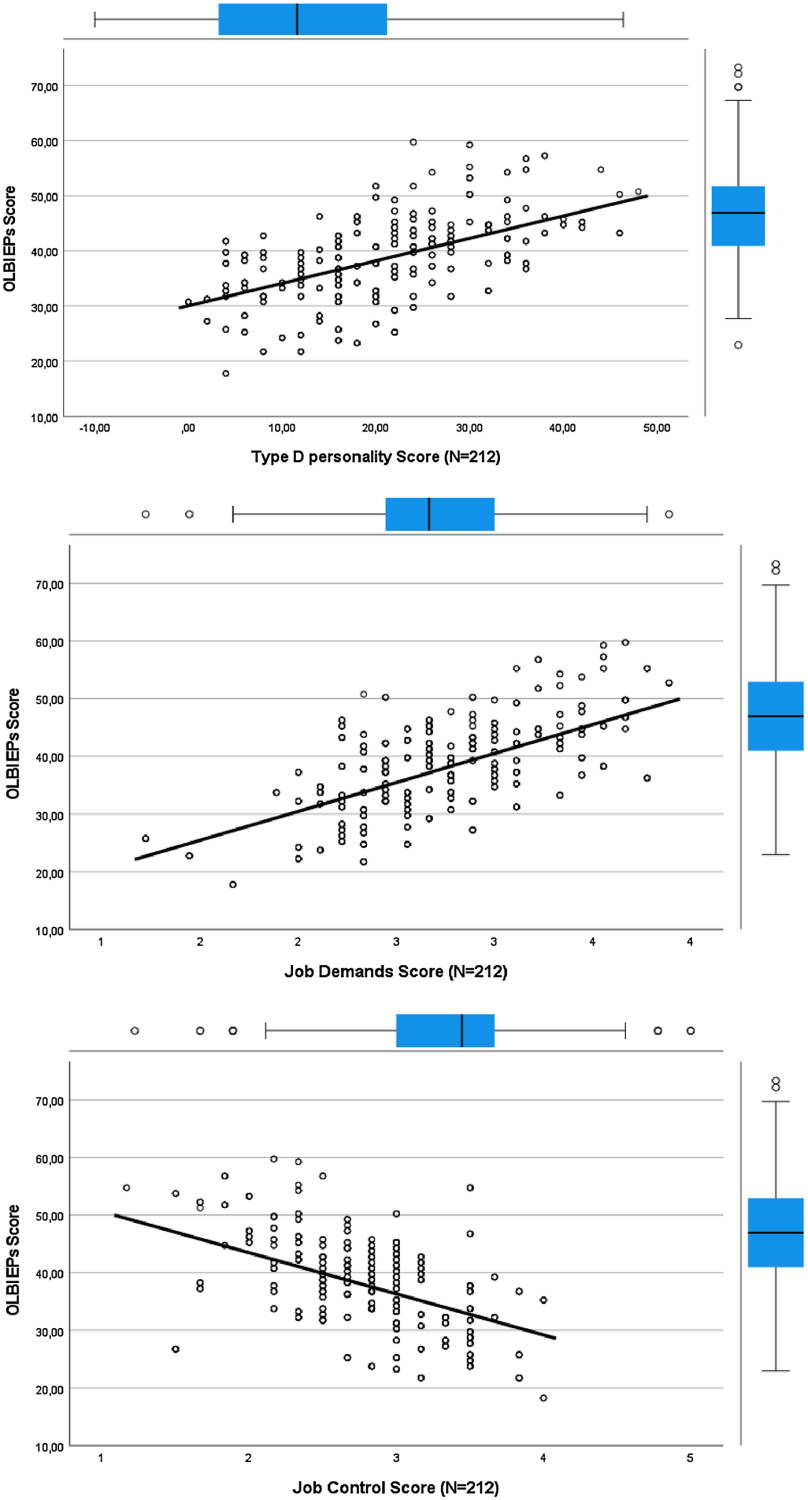
Regression OLBI and Type D personality, OLBI and job demands, OLBI and job control

Therefore, a hierarchical logistic regression analysis was conducted (Table 5) to correct these factors. In the first step we placed the sociodemographic elements as there are age, gender and workregime. We added jobdemands and jobcontrol in step two. We then added support of the supervisor and support of the colleagues in step 3. Finally we added the Type D positive EPs.The relation to the risk of burnout of all the job-related and organizational predictors was calculated. Hence we included these previously mentioned factors in the linear regression analysis in addition to the variable ones comparing emergency physicians with Type D personality to the remainder of emergency physicians of the study group. The regression analysis resulted in Type D, job demands and job control as remaining significant predictors. Most importantly, emergency physicians with Type D were seven times more likely to have a risk of burnout than physicians with non-Type D personalities (OR = 7.82; CI = 3.27 – 18.68). In addition, this aspect explained 23% of the variance in the risk of burnout. The regression OLBI and Type D Personality, OLBI and Job Demands, OLBI and Job Control are more clarified in in the diagrams below Table 5 (Fig. 3).

**Table 5:** Odds ratios for burnout among emergency physicians (fully adjusted logistic regression, N = 212)

	OR	CI	P
Age (per 1 year)	0.63	0.58 – 0.70	0.748
Gender (male vs. female)	0.90	0.66—1.23	0.905
Work regime (full time vs. part time)	1.00	0.70—1.45	0.878
*Job demand (per 1 point increase)*	*3.29*	*1.76—6.16*	*0.013*
*Job control (per 1 point increase)*	*0.19*	*0.08—0.47*	*0.008*
Support supervisor (per 1 point increase)	0.67	0.36—1.27	0.520
Support colleagues (per 1 point increase)	0.56	0.30—1.02	0.207
*Type D personality (positive vs. negative)*	*7.82*	*3.27—18.68*	*0.001*

OR = Odds ratio, CI = 95% confidence interval for OR, p = p value

## Discussion

Emergency physicians showed moderate to high levels of burnout. The study findings indicated difficult work conditions, including significant psychological demands, lack of resources, and poor support. Nevertheless, physicians reported high job satisfaction (Schaufeli et al. 2009; Bragard et al. 2015). These findings suggest that not all physicians exposed to similar job-related and organizational determinants will develop burnout. Furthermore, individual determinants may play an essential role in the development of burnout. Physicians with a Type D personality are seven times more prone to burnout than physicians with another personality type. Type D personality alone explained 22.7% of the variance in the risk of developing burnout. An average of 28.3% to 29.1% of the emergency physicians and hospital specialists in our study showed Type D personality. In contrast, only 21% of the Belgian/Dutch population has been reported to have a Type D personality (Denollet 2005). Additionally, the burnout rate was highest (61.6%) in emergency physicians and similar to observations in previous studies (Rotenstein et al. 2018; Somville et al. 2020). The difference in prevalence in hospital physicians versus emergency physicians may be due to other additional determinants not assessed in this study. Personality types might also influence this variety, since an association was detected between the burnout dimensions and Type D personality (Borkoles et al. 2018; Polman et al. 2010; Oginska-Bulik 2006). As personality is a rather stable trait, it can be argued that Type D personality is risk factor for developing burnout (Denollet 2005). The stability of Type D personality does not mean that a person's level of anxiety and risk of burnout may not be adjustable (Borkoles et al. 2018). Persons with a Type D personality reported poor use of coping strategies, even at lower and average levels of stress, which likely explains their higher levels of perceived stress (Polman et al. 2010; Williams and Wingate 2012). In primary and secondary prevention, adjustment of both social inhibition and negative affectivity is preferred. Consequently, prevention could involve training coping strategies and using positive psychology. Supporting persons or groups can be beneficial in reducing feelings of burnout and tension, especially in emergency physicians with Type D personality. (Oginska-Bulik 2006).

In recent years, emergency physicians have faced additional stress factors, e.g., global warming-associated natural disasters and terror threats, and the recent COVID-19 pandemic; therefore, preventive measures against burnout and sick leave will be crucial in maintaining ED operations.

## Limitations

The number of emergency physicians with Type D personality among this group of physicians is relatively small. Additionally, the prevalence of burnout was calculated using cutoff scores based on Dutch study samples because of the lack of Belgian full scores (Squires et al. 2014). Larger study samples of emergency physicians to confirm and expand our study results are warranted. Self-report questionnaires have limitations. However, the survey did provide noteworthy findings and led to the development of further hypotheses about how Type D personality physicians respond to their work in the ED. We would certainly like to emphasize the generalizability of the conclusions and the fact that the design does not support full causal inferences. Supplementary approaches will be required to entirely test these hypotheses (Spector and Jex 1998).

## Conclusion

We recommend enhanced preventive measures and further coaching programs related to Type D personality to improve the professional well-being of emergency physicians, especially when emergency departments are overwhelmed due to the COVID-19 pandemic and are still facing terror threats. Consequently, we advise the use of preventive measures for emergency physicians who are vulnerable to burnout. A program that includes training coping strategies, aspects of positive psychology and a support group or person might.

## Abbreviations

BeSEDiM: Belgian Society of Emergency and Disaster Medicine
COVID-19: Coronavirus Disease 2019
D: Distressed
DS-14: Distress Scale-14
ED: Emergency department
EPs: Emergency physicians
IC: Intensive care
LQWQ-MD: The Leiden Quality of Work Questionnaire for Medical Doctors
MBI-HSS: Maslach Burnout Inventory Human Service Survey
OLBI: Oldenburg Burnout Inventory
UWES: Utrecht Work Engagement Scale
SPSS: Statistical Package for the Social Sciences
